# The effect of pelvic floor muscle training in men with benign prostatic hyperplasia and overactive bladder

**DOI:** 10.1007/s00345-024-04974-7

**Published:** 2024-05-02

**Authors:** Magdalena Hagovska, Jan Svihra, Ladislav Macko, Jan Breza, Jan Svihra, Jan Luptak, Lubomir Lachvac

**Affiliations:** 1 Department of Physiatry, Balneology, and Medical Rehabilitation, Institution—Faculty of Medicine, PJ Safarik University, Kosice, Slovak Republic; 2 Department of Urology, Jessenius Faculty of Medicine, Comenius University Bratislava, Martin, Slovak Republic; 3 Outpatient Clinic of Urology, Urocentrum, Levice, Slovak Republic; 4 Department of Pediatric Urology, Faculty of Medicine, Comenius University Bratislava, National Institute of Pediatric Diseases, Bratislava, Slovak Republic; 5 Department of Urology, Faculty of Medicine, PJ Safarik University, Kosice, Slovak Republic

**Keywords:** Pelvic floor muscle training, Overactive bladder, Silodosin

## Abstract

**Background:**

Men with overactive bladder (OAB) and benign prostatic hyperplasia (BPH), will have deterioration in the quality of life.

**Objective:**

The aim of this study was to evaluate the effect of combining pelvic floor muscle training with the urgency suppression technique (PFMT-st) and silodosin in comparison with silodosin in men with benign prostatic hyperplasia (BPH) and overactive bladder (OAB) after 12 weeks of treatment.

**Patients and methods:**

A total of 158 patients were randomized into two groups. The control group received oral silodosin at a daily dose of 8 mg. The experimental group was administered PFMT-st and silodosin. The evaluation methods included the number of voids and intensity of urgencies over 24 h using a micturition diary, the International Prostate Symptom Score (IPSS), the Overactive Bladder Questionnaire (OAB-q), and the patient global impression of improvement (PGI-I).

**Results:**

142 of 172 (86.6%) men were assessed (70 in the control group, 72 in the experimental group). The significant changes were in favor of the experimental group (*p* < 0.001) in the number of voids per 24 h (− 1.95 ± 1.94 vs. − 0.90 ± 1.44), the OAB-q symptom score (− 14.25 ± 10.05 vs. − 9.28 ± 10.60), the intensity of urgencies (− 0.97 ± 0.53 vs. 0.24 ± 0.57), the IPSS (− 4.59 ± 3.00 vs. − 2.30 ± 3.63), and in the PGI-I (2.24 ± 0.79 vs. 3.60 ± 0.92).

**Conclusions:**

The addition of PFMT-st to silodosin treatment significantly improved OAB in men with BPH. This is the first study to confirm that PFMT-st should be the first-choice treatment for OAB in BPH.

## Introduction

The International Society for Continence (ICS) and the International Urogynecological Society (IUGA) define overactive bladder (OAB) as urgency with frequent urination and nocturia with or without urgent incontinence in the absence of a urinary tract infection or other disease [1, 2].

Lower urinary tract symptoms (LUTS) are a common problem in adult men and have a significant impact on quality of life. Lower urinary tract symptoms are often related to bladder outlet obstruction (BOO), which most commonly occurs in benign prostatic obstruction (BPO). Bladder prostatic obstruction is often associated with OAB symptoms [3].

The prevalence of male urinary storage symptoms (51%) was higher than that of urinary emptying (25%) and post-micturition symptoms (16%). The overall prevalence of OAB was 11.8% in both sexes and its frequency increased with age. Lower urinary tract symptoms due to benign prostatic hyperplasia (BPH) are common in aging men and deteriorate their quality of life [4, 5].

The choice of LUTS treatment for BPH depends on the clinical findings, treatment efficacy, treatment preferences, and patient expectations in terms of the rate of onset, efficacy, side effects, quality of life, and disease progression. The first choice of drug therapy for male patients with LUTS and BPH is alpha-blockers [4]. If OAB symptoms persist after initial treatment with alpha-blockers, the treatment is supplemented with anticholinergics [6–10].

Silodosin is highly selective for α1A-adrenoreceptors, which occur in the prostate, prostatic urethra, bladder base, and neck. The inhibition of α1A-adrenoreceptors causes smooth muscle relaxation in these tissues and reduces BOO. The aim is to alleviate LUTS caused by BPH [11, 12].

According to the ICS the possible first-choice therapy for OAB is pelvic floor muscle training (PFMT). Pelvic floor muscle training is based on scientific evidence. It is defined as the repeated selective voluntary contraction and relaxation of specific pelvic floor muscles. It is important to train pelvic floor muscles for strength, endurance, and relaxation [13].

Other bladder control techniques include urgency suppression. These methods/maneuvers are used to decrease the feeling of urgency, which may include PFM contraction, distraction of urgency, relaxation, and breathing [13].

As part of monitoring the effect of physiotherapy interventions on OAB, diaphragmatic breathing exercises may be an alternative to PFM exercises for the treatment of urinary incontinence (UI) and OAB [14].

Lifestyle modification is important in the treatment of OAB and may include normal fluid consumption, dietary modification, and physical activity. Other techniques in the treatment of OAB may include bladder training, timed voiding, habit training, and prompt voiding [13–15].

However, studies with better methodological quality are needed to obtain a higher level of scientific evidence and to determine the optimal current modality, type, and parameters for each type of UI and OAB [16, 25]. No studies on the combination of silodosin and PFMT in men with BPH and OAB have been published in the literature.

The aim of our study was to evaluate the effect of combining PFMT with the urgency suppression technique (PFMT-st) and silodosin in comparison with silodosin alone in men with BPH and OAB after 12 weeks of treatment.

## Patients and methods

### Study design

The SILODOSING study was a randomized controlled study with a 1:1 allocation that compared the effects of silodosin treatment and combination therapy with silodosin and PFMT-st in patients with BPH and OAB. The protocol was approved by the Local Ethics Committee. This study was conducted from January 2021 to September 2022. All men received written information about the study and signed informed consent, and urologists from Slovak urological outpatient clinics enrolled them according to the inclusion and exclusion criteria.

### Allocation

**Fig. 1:**
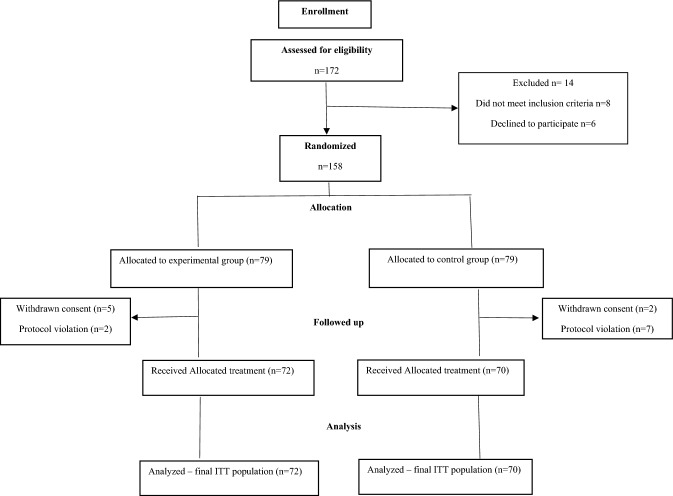
CONSORT diagram

The SILODOSING study was performed at 21 urological outpatient clinics at a national level. Patients were randomized into two parallel groups: experimental group (E) and control group (C). This was a randomized controlled study with a 1:1 allocation ratio. Computer-generated sequences were used in the allocation process by a researcher who did not participate in the study. The computer-generated even and odd numbers of the patients. Odd-numbered patients continued with silodosin monotherapy at a dose of 8 mg once daily for 12 weeks, while even-numbered patients continued treatment with silodosin at a dose of 8 mg once daily and had PFMT-st treatment added at the same time. The generated numbers were then placed in sealed envelopes. Each envelope contained the codes for the experimental and control groups. (Fig. 1). Data collection was conducted anonymously. The training staff was not blinded, but the assessors were blinded to the intervention (Table 1).

**Table 1:** Recruitment, intervention, and evaluation plan for experimental and control groups in the SILODOSING study

Time period	Enrollment (T0) before treatment (T1)	After 12 weeks of treatment (T12)
Informed consent	x	
Enrollment	x	
Allocation	x	
Demographic data	x	
Voiding diary	x	x
The International Prostate Symptom Score (IPSS)	x	x
Overactive Bladder Questionnaire (OAB—q)	x	x
The Patient Perception of Intensity of Urgency Scale (PPIUS)	x	x
Patient Global Impression of Improvement score (PGI-I)		x
Adverse events—harms	x	x
Adherence	x	x
Intervention for experimental group	x	x

### Sample size estimation

We used an estimate made by sampling the number of patients based on a 0.80 power of test and 0.05 alpha (type I error of). Using a statistical program, we calculated the number of patients with an expected improvement in IPSS symptoms of > 25%. We recruited 63 participants in the experimental group and 63 in the control group. We expected 20% loss, resulting in the inclusion of at least 158 patients.

### Inclusion criteria

The inclusion criteria were as follows: willing to provide written informed consent; men over 50 years of age with LUTS, OAB, and BPH; persistence of OAB after four weeks of silodosin treatment; symptoms of OAB (urinary frequency and urgency with or without urinary incontinence) for ≥ 3 months before visit 1; willing and able to complete the 3-day voiding diary and questionnaires; an International Prostate Symptom Score (IPSS) ≥ 8; experiencing an average of eight or more micturitions per day over a 3-day diary period; and experiencing an average of two episodes of urgency per day (grade 3 or 4) over a 3-day diary period.

### Exclusion criteria

The exclusion criteria were as follows: Post-void residual volume (PVR) > 200 mL. Evidence of urinary tract infection and hematuria. Use of anticholinergics or beta 3 mimetics within 4 weeks prior to visit 1 and during the study. Oncological diseases of the lower urinary tract and prostate. Neurogenic bladder. Urethral strictures and bladder neck stenosis. Urolithiasis. Diabetes mellitus. Previous lower urinary tract surgery Stress urinary incontinence. Intermittent catheterization. Chronic urinary tract infection. Previous Botox treatment in the last 12 months. Chronic electrostimulation treatment of OAB in the last 12 months. The patient began or had changed bladder training programs or pelvic floor exercises for less than 90 days before enrollment (T0). Cognitive deficits and dementia. Interventional trial within 30 days prior to enrollment (T0). The total daily urine production was over 2500 mL according to the voiding diary.

### Procedures

#### Experimental and control groups

Continuation of silodosin treatment at a dose of 8 mg daily for 12 weeks.

##### Experimental group

Peroral treatment with silodosin at a dose of 8 mg daily.

1. Standard physiotherapeutic assessment including history and physical examination and investigations. ^13^
2. The PFMT intervention was performed by a trained physiotherapist who explained the correct exercise technique.
3. If the patients were able to perform PFM contractions correctly, they were allowed to continue with the exercises at home (Table 1).
4. PFMT was conducted for 30 min/day, 5 days/week for 12 weeks, and supervised by a physiotherapist once a week.

*Intervention*: Pelvic floor muscle training with urgency suppression techniques (PFMT-st).

Education:

1. Education about proper urination training.
2. Education on the principle and effect of PFMT.
3. Education about proper ergonomy during everyday activities.
4. Education about the correct breathing pattern and stereotype.

Exercises:

1. Exercises for PFM awareness.
2. Exercises to improve strength and endurance of the PFM.
3. Exercises for PFM relaxation.

(Table 2).

**Table 2:** Exercise program for men with BPH and OAB

	Description
1	Education about proper urination training Education about proper urination training in the period without pathological urgency. Education about conscious urgency suppression through PFMT—the suppressive urgency technique Education about healthy urge to urinate and pathological urge to urinate Education on how to cancel a sudden, urgent urge to urinate: Tighten the pelvic floor muscles and pull them as if inside the body and hold on for 5 s, then release them and continue until the urinary urgency retreated
2	Education on the principle and effect of PFMT Do not contract m. gluteus maximus, m. rectus abdominis, and adductors instead of the pelvic floor muscles The principle of exercise: With expiration, tighten the pelvic floor muscles and pull them as if inside the body and hold on for 5 s, then release them
**3**	Education about proper ergonomy during everyday activities Education about proper ergonomy during everyday activities—correct sitting, standing, walking, lifting, bending
**4**	Education about correct breathing pattern and stereotype When lying on your back or sitting, keep your chest in an exhaled position With inhalation to the area of the lower ribs, you activate your diaphragm With exhalation, pull the navel toward the spine to activate the deep abdominal muscles
**5**	Exercise Phase 1: Weeks 1 and 2 Exercise 1: Pelvic floor muscle awareness Exercise positions: Lying on the back with lower limbs bent, lying on the abdomen, then sitting, standing Exercise: Awareness of the pelvic floor muscles During inspiration, imagine that you want to draw the pelvic floor muscles inside the body, and then relax the pelvic floor muscles during expiration, both in the rhythm of normal breathing. Repeat 10–20 times
**6**	Exercise Phase 2: Weeks 3–12 Exercise 2: Exercises to strengthen the pelvic floor muscles Exercise positions: Lying on the back, lying on the abdomen, then sitting, standing, and walking (medium and very strong contraction of pelvic floor muscles) Exercise: a. With expiration, tighten the pelvic floor muscles and moderately pull them into the body for 10 s, then relax for 10 s. Repeat 10 times in each position b. With expiration, tighten the pelvic floor muscles and pull them into the body very hard for 5 s, then relax for 5 s. Repeat 10 times in each position
**7**	Exercise 3: Pelvic floor muscle relaxation exercises Exercise positions: Lying on the back, lying on the abdomen, then sitting Exercise: In normal breathing rhythm a. Tighten the pelvic floor muscles moderately for 1 s and then relax for 10 s b. Tighten the pelvic floor muscles slightly for 5 s and then relax for 10 s

### Outcome measures

#### Primary outcomes

Changes in the number of voids and intensity of urgencies over 24 h using a voiding diary. The voiding diary included voided volume over 24 h (mL), number of voids per 24 h, voided volume during the day (mL), number of voids per day, voided volume during the night (mL), nighttime frequency (nocturia), and mean voided volume per 24 h, during the day, and during the night (mL) for 3 days [1]. The Patient Perception of Intensity of Urgency Scale (PPIUS) evaluates the severity of symptoms of OAB: 0 = no urgency, 1 = mild urgency, 2 = moderate urgency, 3 = serious urgency, and 4 = urgency urinary incontinence [17]. OAB is defined as urgency one or more times a day, voiding eight or more times a day, nocturia two or more times at night, with or without urgency UI [1].

#### Secondary outcomes

The International Prostate Symptom Score (IPSS) is a 7-point scale for assessing BPH symptoms: ratings 0–7 mildly symptomatic, 8–19 moderately symptomatic, and 20–35 severely symptomatic [18, 19]. Overactive Bladder Questionnaire (OAB-q) focused on the symptoms of OAB incontinence in the last four weeks (0 without symptoms, 100 most symptoms) and quality of life (100 best quality of life, 0 worst quality of life) [20–22]. The Overactive Bladder Symptom Score (OABSS) comprises seven questions and five answers. The total score was 0 (no symptoms) and 28 (severe symptoms) [23]. Patient Global Impression of Improvement score (PGI-I) was compared with the condition before the patient started treatment in the study: scores 1 = much better, 2 = quite better, 3 = a little better, 4 = no change, 5 = a little worse, 6 = much worse, and 7 = much worse.

Incidence of adverse events.

Treatment and serious side effects were monitored during the entire clinical trial period [24].

### Statistical analysis

The intention-to-treat (ITT) population consisted of all subjects who had primary and secondary outcome measures. Descriptive and inferential statistics were used for data analysis. Unpaired t-tests were used to compare the experimental and control groups before the training. Our data showed a normal distribution. Differences between the control and experimental groups before and after the intervention were evaluated using a general linear model (GLM), mixed design ANOVA, and repeated measurements with Greenhouse–Geisser correction. The level of statistical significance was set at *P* < 0.05. The effect size (ES) was calculated based on the partial eta squared (η2). The specification for ES was small, medium, and large effect sizes and classified as η2:0.00–0.003, no effect size; η2:0.010–0.039, small effect size; η2:0.060–0.110, medium effect size; and η2:0.140–0.200, large effect size. Calculations were performed using IBM SPSS Statistics for Macintosh, Version 27.0 (Armonk, NY: IBM Corp.)

## Results

**Fig. 2:**
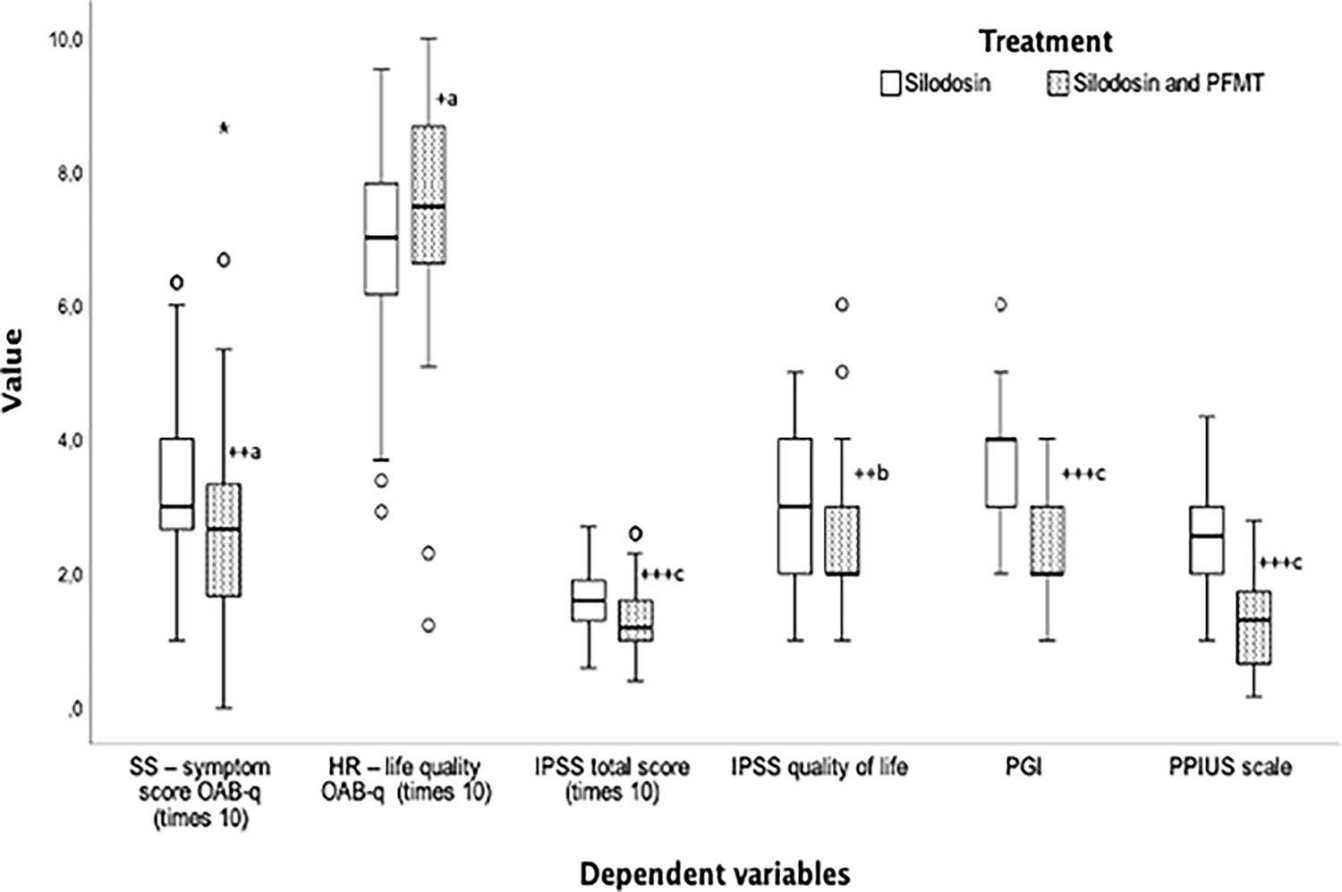
Statistical comparison between groups after 12 weeks of treatment. p-value mixed design analysis of variance (ANOVA) after treatment between groups. (^+^*p* < 0.05; ^++^*p* < 0.01; ^+++^*p* < 0.001). Effect size (ES) will be calculated based on partial eta squared. The small, medium, and large effect sizes (η2) will be classified as 0.00–0.003, no effect; 0.010–0.039, small; 0.060 to 0.110, medium; 0.140–0.200, large (Cohen, 1988). (^a^small; ^b^medium; ^c^large)

Of 172 patients, 14 (%) were excluded because they did not meet the inclusion criteria. A total of 158 patients were randomized into two groups: 79. Seven men were excluded from the experimental group because they did not meet the ITT criteria. In the control group, nine men were excluded because they did not fulfill the ITT principle. The final group consisted of 70 men in the control group with a mean age of 68.96 ± 9.71 years and 72 men in the experimental group with a mean age of 66.25 ± 7.94 years (Fig. 1). No significant differences were observed in the demographics and pre-treatment parameters between the groups in age, body mass index, symptom score, quality of life in the OAB questionnaire, parameters of the micturition diary, PPIUS score, IPSS symptom score, and quality of life (Table 3). The final significant differences were noted in favor of the experimental group (*p* < 0.001), the majority of monitored parameters with small–medium ES. There were no significant differences between the groups in voided volume during 24 h, day, or night (Table 4). The significant differences were noted in favor of the experimental group (*p* < 0.001) in the OABSS and quality of life, the PPIUS in 24 h, the IPSS score, and in the PGI-I with small ES. There were no significant differences in the nocturnal polyuria index between the groups. After treatment, within the experimental and control groups, a significant improvement was noted in all the monitored parameters (Fig. 2). Before OAB treatment, the following adverse events were observed: tachycardia, nasal congestion, dizziness, nausea, dry mouth, skin rash, and ejaculatory disorder. Dizziness and ejaculatory disorders occurred most frequently (Table 5).

**Table 3:** Demographic and baseline characteristics of study participants

	1 Mean ± SD	2 Mean ± SD	p
N	72	70	
Age	66.25 ± 7.94	68.96 ± 9.71	0.07
Body mass index (kg/m^2^)	28.58 ± 5.06	28.82 ± 3.85	0.95
SS – symptom score OAB-q	40.93 ± 11.98	42.52 ± 13.86	0.46
HR – life quality OAB-q	61.22 ± 14.50	61.54 ± 15.39	0.89
Number of voidings per 24 h	10.60 ± 1.96	10.54 ± 1.89	0.86
Number of voidings per day	8.26 ± 1.54	8.16 ± 1.60	0.68
Number of voidings per night	2.46 ± 0.97	2.37 ± 0.92	0.58
Voided volume during 24 h, mL	1705.10 ± 347.74	1700.29 ± 317.11	0.93
Voided volume during day, mL	1352.42 ± 310.49	1349.86 ± 272.95	0.95
Voided volume during night, mL	352.68 ± 143.85	350.43 ± 170.03	0.93
Mean voided volume per 24 h, mL	165.74 ± 42.78	165.34 ± 38.03	0.95
Mean voided volume during day, mL	168.87 ± 47.03	170.38 ± 40.78	0.83
Mean voided volume during night, mL	153.39 ± 65.33	155.29 ± 64.19	0.86
Nighttime polyuria index	0.20 ± 0.07	0.20 ± 0.08	0.80
Patient Perception of Intensity of Urgency Scale	2.25 ± 0.38	2.21 ± 0.47	0.58
IPSS total score	17.32 ± 4.27	18.51 ± 5.07	0.13
IPSS quality of life	3.53 ± 1.05	3.51 ± 1.24	0.94

p- t-test

1—silodosin and PFMT-st, 2—silodosin

**Table 4:** Statistical comparison of dependent variables between groups after 12 weeks of treatment

	SK	Mean ± SD T1	Mean ± SD T2	p	Partial eta squared (η2)	Interpretation
Number of voidings per 24 h	1	10.60 ± 1.96	8.46 ± 2.24	0.003	0.061	medium
	2	10.54 ± 1.89	9.46 ± 1.66			
Number of voidings per day	1	8.26 ± 1.54	7.15 ± 1.88	0.024	0.036	small
	2	8.16 ± 1.60	7.79 ± 1.38			
Number of voidings per night	1	2.46 ± 0.97	1.49 ± 0.97	0.016	0.041	small
	2	2.37 ± 0.92	1.86 ± 0.82			
Voided volume during 24 h, mL	1	1705.10 ± 347.74	1654.93 ± 326.89	0.851	0.000	no effect
	2	1700.29 ± 317.11	1665.60 ± 348.45			
Voided volume during day, mL	1	1352.42 ± 310.49	1395.76 ± 322.65	0.353	0.006	no effect
	2	1349.86 ± 272.95	1346.23 ± 310.81			
Voided volume during night, mL	1	352.68 ± 143.85	259.17 ± 148.62	0.016	0.041	small
	2	350.43 ± 170.03	319.37 ± 144.02			
Mean voided volume per 24 h, mL	1	165.74 ± 42.78	199.93 ± 47.31	0.003	0.06	medium
	2	165.34 ± 38.03	176.97 ± 43.93			
Mean voided volume during day, mL	1	168.87 ± 47.03	203.12 ± 50.59	0.001	0.078	median
	2	170.38 ± 40.78	176.25 ± 42.23			
Mean voided volume during night, mL	1	153.39 ± 65.33	190.11 ± 61.35	0.451	0.004	no effect
	2	155.29 ± 64.19	181.58 ± 66.64			

T1, T2; Time 1—baseline, Time 2 after 12 weeks. 1—silodosin and PFMT-st, 2—silodosin

p-value mixed design analysis of variance (ANOVA) after treatment between groups. Effect size (ES) will be calculated based on partial eta squared (h2). According to Cohen, the small, medium, and large. effect sizes (η2) will be classified as 0.00–0.003, no effect; 0.010–0.039, small; 0.060–0.110, medium; 0.140–0.200, large (Cohen, 1988)

**Table 5:** Incidence independent variables—silodosin adverse events before OAB treatment

	1(%)	2(%)
Tachycardia	1.4	5.7
Nasal congestion	4.2	5.7
Dizziness	9.7	10
Nausea	2.8	4.3
Dry mouth	4.2	10
Skin rash	1.4	0
Ejaculatory disorders	6.9	5.7

1—silodosin and PFMT-st, 2—silodosin, p: chi-square test = no significant differences between groups

## Discussion

The aim of our study was to evaluate the effect of a combination of PFMT and silodosin compared to silodosin alone in men with BPH and OAB after 12 weeks of treatment.

There were no significant differences between the groups in terms of demographics or baseline parameters. After 12 weeks of treatment, significant differences in all parameters of the micturition diary, in the OABSS and quality of life, the IPSS, and in the PGI-I score were observed in favor of the experimental group. The adverse events in our study included dizziness, tachycardia, nasal congestion, nausea, dry mouth, skin rash, and ejaculatory disorders. Dizziness was the most common adverse effect observed.

Chapple et al. [6] compared the effects of 12 weeks of treatment with alpha-blockers in the control group and tolterodine and alpha-blockers in the experimental group in 652 men with OAB. The results in the micturition diary parameters in the number of voids per 24 h (− 1.8 vs. − 1.2) were in favor of the experimental group. Kaplan et al. [8], in a study of 879 patients with BPH and OAB, compared the effects of placebo, tolterodine, tamsulosin, and tolterodine with those of tamsulosin. There were significant differences in the number of voids per 24 h in the group of patients treated with tamsulosin and tolterodine compared with those in the placebo (− 2.54 vs. − 1.41). In comparing the parameters of the micturition diary, we found the following differences compared to other studies: a decrease in the number of voids per 24 h in the experimental group compared to the control group (− 1.95 vs. − 0.90), mean voided volume per 24 h increased by about 30.99 in the experimental group and in 18.56 in the control group; the Patient Perception of Intensity of Urgency Scale score decreased by − 0.97 in the experimental group and increased by + 0.24 in the control group.

Chapple et al. [6], in the same study (alpha-blockers in the control group and tolterodine and alpha-blockers in the experimental group), found superiority of the combination treatment in the experimental group in reducing OABSS (− 17.9 vs. − 14.4). Matsukawa et al. [9] compared the added effect of fesoterodine and mirabegron in 120 patients with persistent OAB when treated with silodosin in BPH patients for 12 weeks. The fesoterodine group had a more significant decrease in OABSS compared with the mirabegron group (− 2.8 vs. − 1.5), as well as a decrease of IPSS (− 4.1 vs. − 3.8). Matsukawa et al.^10^ compared monotherapy with silodosin and naftopidil in 314 men with OAB and BPH for 12 weeks. The silodosin group vs. the naftopidil group showed a significant decrease in the OAB symptom-specific questionnaire score (− 2.8 vs. − 2.3). We found significant differences in the OAB-q scores compared with other studies. There was a reduction in OABSS in the experimental group vs. the control group (− 14.25 vs. − 9.28) and an increase in the quality of life score (12.84 vs. 7.34).

The IPSS quality of life improved in both groups (− 1.5 vs. − 1.1). Kaplan et al. [8] found significant differences in IPSS scores in the tamsulosin group with tolterodine compared to placebo in favor of the treatment group (− 8.02 vs. − 6.19), and in quality of life (− 1.61 vs. − 1.17). According to the IPSS, we found the following differences compared to other studies: a decrease in the total IPSS score in the experimental group vs. in the control group (− 4.59 vs. − 2.30) and an improvement in quality of life (− 1.13 vs. − 0.63).

## Strengths of the study and its limitations

The strength of this study is that it promotes non-invasive and conservative treatment (PFMT-st), in addition to silodosin treatment. The combination treatment can significantly reduce OAB symptoms in patients with BPH, reduce the incidence of adverse effects after other OAB treatments, and encourage it to be used in the first-line treatment.

The limitations are the minimum recommended duration of PFMT (only 12 weeks) and the non-use of invasive urodynamic examinations.

## Conclusions

The addition of PFMT-st to silodosin treatment significantly improved OAB in men with BPH compared to silodosin alone. The PFMT-st was perceived positively, motivated the participants, and had good adherence, compared to those of other randomized controlled clinical drug treatments.

## Recommendations

1. Increase the number of patients in each group.
2. Prolong the follow-up period of patients.
3. Use combination therapy in patients with ED.

## Data Availability

We have not received ethical approval to share raw clinical trial data. The data that will be collected will be available upon request to the author.
